# Supplementary material for: Evaluation of plasma viral-load monitoring and the prevention of mother-to-child transmission of HIV-1 in three health facilities of the Littoral region of Cameroon
Source: PLoS One. 2022 Nov 7;17(11):e0277271. doi: 10.1371/journal.pone.0277271 (PMC9639847; doi:10.1371/journal.pone.0277271)
Supplement: Attachment — Submitted filename: Comments-Section by section-DK.docx [file pone.0277271.s001.docx]

**General:**

Overall, the manuscript presents evidence that can assist to improve the PMTCT program in the study HFs in Cameroon.

**Major comments:**

1) About the study design(Line 94-95), ….. a hospital-based cohort-study..

- **It is known that cohort study is a type of longitudinal study,** which is an approach that follows research participants over a period of time (often many years).
- But in this study, mother-child couples **(the cohort)** were scheduled for two monitoring assessments (the first assessment was **after delivery** and the second one **at six weeks after delivery**). And then, the **primary outcomes** (VL monitoring, mode of delivery according to VL and early infant diagnosis result) were measured **only once,**  **at six weeks.** So, as a cohort study, there are no multiple measurers (every two week, three month, ..) of the primary outcomes, especially VL coverage, has been done at defined time intervals
- Hence, you need to define how the follow up was done between 1998-2000 and what parameters (the primary outcomes, or other parameter) has been measured at regular intervals. And you need to show the results in Tabe form. **Otherwise, this study might not qualify the study design as “Cohort study”**

2) In the result section (Line 199 – 216), There is/are no results (Tables) which support the explanation

**Minor comments:** showed in the Table below (in attachment)

**Section by section questions/Comments/suggestions**

| **Section** | **Line No and Concerns highlighted in red font** | **Questions/Comments/suggestions** |
| --- | --- | --- |
| Title |  |  |
| Abstract-**Result** | Line 36-37: VL-coverage during antenatal care (ANC) was 50.4% (68/135), with a lower VL coverage before the year-2020 (37.5% v. 61.9%, p=0.0069). | ….before the year 2020 … is an open end. Could it be with the study period (between 2019 to 2022)? |
|  | Line 37-38: Married women (p=0.0273) and those on treatment before pregnancy (p<0.0001) had a higher VL coverage. | - Indicate the comparison group in each: Married women vs…; those on treatment vs…. - “Higher” is subjective --- better to be supported with figures (%) |
|  | Line 40: … suggesting an overall viral suppression of 89.7% (61/68). | - … over all viral suppression…… can you include (<= 1000 copies/ml) |
| **Conclusion** | Line 45-46: In these Cameroonian settings, VL-coverage remains **suboptimal** among ANC attendees, | - Your reference? National coverage, national target, or global target? Please indicate (? %) |
|  | Line 46-47: Viral suppression rate remains below the target for eliminating MTCT | - …. Below the target for eliminating MTCT ….. national or global target, please specify, and indicate the target in bracket (? %) |
|  | Line 48-49: Thus, achieving an optimal PMTCT performance requires a thorough compliance to virologic assessment during ANC. | - This conclusion needs to be supported by findings showed in the result section. i.e. Was routing VL testing for the HIV Pos pregnant women has not been done as per the national/global guideline during the study period-2019-2022 ? |
| Study population | Enrolment of study participants | Any exclusion criteria? Please indicate |
|  |  |  |
| Results | Line 200-201…with a lower VL-coverage before the year-2020 | Please qualify “Lower” number ? (%); and also qualify “before the year 2020”, does it mean between 1998-2000 ? |
|  | Line 201-202: …..Married women (p=0.0273) and those on treatment before pregnancy (p<0.0001) had a higher VL-coverage | Please show, with whom the “Married women” and those on treatment” are compared and indicate the figures (%) like ---% vs ….. (p=0.0273, for both comparisons |
|  | Line 199-2016 …. | There is/are no results (Tables) which support the explanation |
| Discussion | Overall, the results are well discussed!! | |
|  | Line 242 … regarding your discussion on VL coverage, you need to show your results in Table in the result section, then you can discuss your findings. | Overall, you better show in Table (s) of the results regarding VL coverage over time (during the study period) |
|  | Line 246-247 ….Unlike recommendations from our national guidelines on PMTCT | Pleas show what the national guideline recommends |
| Conclusion | Line 324 ..Nonetheless, there is an improvement in scaling-up VL overtime, | As commented earlier, VL coverage overtime need to be showed in the result section in a Table |
